# Transactions of the Medical Association of Southern Central New York, at the Annual Meeting Held at Elmira, June, 1850

**Published:** 1850-09

**Authors:** 


*Transactions of the Medical Association of Southern Central New York, at the Annual Meeting held at Elmira, June,* 1850.

We have read these Transactions with much interest. They evince an ardent devotion to the cause of medical science, in the practical part of its duties, and show a highly commendable spirit. The most interesting features of these Transactions are the reports on the endemics and epidemics, of the various counties, embraced within the society. A great variety of topographical, and etiological information is thus presented to the profession, and contributes to the general interests of medical science. .B

